# Prevalence and intensity of *Loa loa* infection over twenty-three years in three communities of the Mbalmayo health district (Central Cameroon)

**DOI:** 10.1186/s12879-019-3776-y

**Published:** 2019-02-13

**Authors:** Aude E. Mogoung-Wafo, Hugues C. Nana-Djeunga, André Domche, Floribert Fossuo-Thotchum, Jean Bopda, Steve Mbickmen-Tchana, Honoré Djomo-Kamga, Joseph Kamgno

**Affiliations:** 1 Centre for Research on Filariasis and other Tropical Diseases, PO Box 5797, Yaounde, Cameroon; 2 Parasitology and Ecology Laboratory, Department of Animal Biology and Physiology, Faculty of Science, University of Yaoundé 1, Yaoundé, Cameroon; 3 Faculty of Medicine and Biomedical Sciences, University of Yaoundé 1, PO Box 1364, Yaounde, Cameroon

**Keywords:** Loiasis, Prevalence, Intensity of infection, Mbalmayo health district, Cameroon

## Abstract

**Background:**

Loiasis is a vector-borne parasitic disease due to *Loa loa* and transmitted to humans by tabanids of the genus *Chrysops*. Loiasis has been historically considered as the second or third most common reason for medical consultation after malaria, and a recent study has reported an excess mortality associated with the infection. However, the clinical impact of this filarial disease is yet to be elucidated, and it is still considered a benign disease eliciting very little attention. As a consequence of post-treatment severe adverse events occurring in individuals harboring very high Loa microfilarial loads, ivermectin is not recommended in onchocerciasis hypo-endemic areas that are co-endemic for loiasis. Without treatment, it is likely that the transmission of the disease and the morbidity associated with the infection will increase over time. This study aimed at investigating the long-term trends in prevalence and intensity of *Loa loa* infection in an area where no mass anti-filarial treatment has ever been distributed.

**Methods:**

A cross-sectional survey was conducted in three communities of the Mbalmayo health district (Central Cameroon). All volunteers, males and females aged five years and above, underwent daytime calibrated thick blood smears (CTBS) to search for *L. loa* microfilariae (mf). A structured questionnaire was administered to assess the history of both loiasis related clinical signs and migration of enrollees.

**Results:**

The prevalence of loiasis was 27.3% (95% CI: 22.3–32.9) in the three surveyed communities, with a mean mf density of 1922.7 (sd: 6623.2) mf/mL. *Loa loa* infection rate was higher amongst females than in males (*p* = 0.0001) and was positively associated with age of (OR = 1.018; *p* = 0.007). The intensity of infection was higher among males than in females (*p* < 0.0001), and displayed a convex in form trends with age groups. The follow up over 23 years revealed that both the rate and intensity of infection were similar to baseline.

**Conclusions:**

The prevalence and intensity of *Loa loa* infection 23 years on is stable over time, indicating that this filarial disease might be noncumulative as regarded till now.

## Background

Loiasis, better known as the African eye worm, is a parasitic disease caused by the filarial nematode *Loa loa* and transmitted to humans by tabanids belonging to the genus *Chrysops*. *Chrysops dimidiata* and *C. silacea*, commonly known as deer flies and mango or mangrove flies, are the two main anthropophilic vector species, only found in Africa [1]. The disease is restricted to western and central Africa where a geostatistical analysis of history of eye worm has mapped high risk levels of loiasis in 10 countries where an estimated 14.4 million people live [2]. Although most cases of loiasis are asymptomatic [3], the most striking clinical manifestations of this filarial infection are the migration of the adult worm under the bulbar conjunctiva, and the migratory transient edema called Calabar swellings. In addition, loiasis has been historically ranked as the second or third most common reason for medical consultation after malaria and pulmonary diseases in some communities of Congo and Gabon [4, 5]. Data reporting implication of loiasis on human health are very scanty, and its clinical impact is yet to be clarified [6]. Loiasis is still considered a benign disease eliciting very little attention, although a recent survey has reported an excess mortality associated with the infection [7].

Due to the benign nature attributed to this tropical disease, no large-scale control program is dedicated to loiasis though four chemotherapeutic agents (diethylcarbamazine, ivermectin, albendazole and mebendazole) have been proven effective against the parasite [6]. However, it is now accepted that the most effective drugs on *L. loa* infection are not really safe when individuals are heavily infected, and the safer ones are not completely efficient [6, 8]. Indeed, although benzimidazoles are safer for the treatment of loiasis, their effects on microfilarial loads are quite modest or transitory [8, 9]. In contrast, diethylcarbamazine and ivermectin are very efficient against *L. loa*, but therapeutic accidents with sometimes fatal outcomes have been reported in some individuals harboring high microfilarial loads (>8000 microfilariae per milliliter, mf/mL) after treatment with each of these two drugs [10].

In areas endemic for loiasis and where onchocerciasis is meso- (> 40% mf or > 20% nodule prevalence) or hyper-endemic (> 60% mf or > 40% nodule prevalence), ivermectin treatments have been recommended since the clinical impact and consequences associated to onchocerciasis outweighed the risk of post-treatment severe adverse events (SAEs), under specific surveillance system for early detection and management of SAE cases [11]. As a consequence of the collateral impact of ivermectin on *Loa loa*, the transmission of infection has been reduced in some areas under community directed treatment with ivermectin (CDTI) [12]. However, ivermectin is not recommended in hypo-endemic areas for onchocerciasis (< 40% mf or < 20% nodules prevalence) that are co-endemic for loiasis [11]. One can anticipate an increase in transmission and morbidity as well as life expectancy reduction of infected individuals [7].

The objective of the present study was to follow up the prevalence and intensity of *Loa loa* infection over 23 years in three neighboring communities of the Mbalmayo health district (Central Cameroon) in order to investigate the long-term trends in infection parameters in an area where no mass anti-filarial treatment was ever distributed.

## Methods

### Study area and population

This study was conducted in the Mbalmayo health district (Nyong-et-So’o Division, Centre Region) located at about 50 km south of Yaoundé, the political capital city of Cameroon. In this forested environment, the Nyong River is the main watercourse of the study area. Together with its numerous tributaries, this low flowing river generates swamps where raffia grows, thus providing suitable breeding sites for chrysops. The mean annual rainfall is 1600 mm and the mean annual temperature is 24.1 °C.

The population of this area is mainly made of Beti. The major occupations of the populations are subsistence agriculture and cocoa cultivation. The urbanization of the Mbalmayo town, head of the Nyong-et-So’o division, has generated many activities attracting populations of the neighboring villages, especially youth.

### Study design

The study area, known to be endemic to loiasis [2, 13, 14], has sheltered a clinical trial in 1993, aiming at determining whether large-scale ivermectin treatment would have an impact on the transmission of loiasis [15]. Baseline data on loiasis were thus collected, and the present study aimed to investigate the prevalence and intensity of *Loa loa* infection over 23 years. To do this, a cross-sectional survey was conducted in three communities of the Mbalmayo health district (Central Cameroon) to assess the prevalence and intensity of *Loa loa* infection. Eligible individuals were both males and females, aged 5 years and above, who had already lived in the selected community for at least 5 years. All volunteers underwent blood sample collection to search for *Loa loa* microfilariae, and a structured questionnaire was administered to assess the history of both loiasis related clinical signs and migration of populations.

### Sample collection and processing

From each participant, a calibrated thick blood film was collected daytime (from 10 am to 4 pm) to take into account the diurnal periodicity of *Loa loa* [16]. Trained and qualified lab technicians collected a 50 μL sample of finger-prick blood from each study participant using a non-heparinized capillary tube. Blood samples were collected in aseptic conditions using sterile and disposable equipment. Standard procedures were used for blood samples processing and analysis [17]. Slides were examined independently using bright field microscopy (magnification × 10 and × 40 for identification) by two experienced laboratory technicians. All microfilariae present in the blood sample were identified and counted, and the results expressed as microfilariae per mL of blood (mf/mL) [17].

### Identification of Loiasis-related clinical signs

The most striking clinical manifestations attributable to loiasis were assessed using a standardized questionnaire adapted to the proven Rapid Assessment Procedure for Loiasis (RAPLOA) based on the history of eye worm or Calabar swellings [18]. Indeed, the questions asked to the participants were “Have you ever experienced or noticed worms moving along the white of the lower part of your eye?” to assess the experience of eye worm, and “Have you ever experienced swellings under the skin which changed position and disappeared?” to investigate the experience of Calabar swellings. Investigators were assisted by local guides to ease communication with villagers, especially illiterates.

### Migration history

A brief questionnaire was administered to all survey participants to check whether they were foreigners or natives of the targeted community. The number of years of residence in the selected community was recorded and foreigners or visitors were asked where they were originating from in order to identify the origin of the disease in case of infection.

### Data analysis

All relevant data for loiasis were recorded into a purpose-built Microsoft Access database and exported into PASW Statistics version 18 (SPSS Inc., Chicago, IL, USA) for statistical analysis. Microfilariaemia and clinical sign prevalence were expressed as the percentage of infected or affected individuals (presenting with microfilariae in their blood or having experienced eye worm migration or Calabar swelling) among the total number of individuals examined; the 95% confidence interval (CI) was calculated using the Wilson method not corrected for continuity [19]. The intensity of infection was computed when the microfilarial count was available as arithmetic means, and the sampling fluctuations estimated using standard deviation (sd). Chi-square, Mann-Whitney and Kruskal-Wallis tests were used to compare loiasis prevalence and mean intensity of infection between communities, genders and age groups, respectively.

## Results

The survey was carried out in three small neighboring communities (Abang, Akometam and Ngat-Bane) of the Mbalmayo health district. A total of 271 individuals were examined, among whom 140 (51.6%) were females. The median age of the population was 50 years old (interquartile range: 28–63).

### Prevalence

Among the 271 individuals examined, 74 (27.3%; 95% CI: 22.3–32.9) were harboring *L. loa* microfilariae in their blood. Although the prevalence of loiasis was similar among communities visited (Chi-square: 1.604; *p* = 0.448), a high heterogeneity was observed in Loa distribution between genders and age groups (Table 1). Loiasis was more prevalent in females as compared to males (Chi-square: 13,027; *p* < 0.0001), and was gradually increasing in prevalence between age classes (Table 1). Indeed, a positive correlation was observed between infection with *Loa loa* and the age of enrollees (OR: 1.018; 95% CI: 1.005–1.032; *p* = 0.007).

**Table 1:** Prevalence and intensity of loiasis in three neighboring communities of the Mbalmayo health district

Variables	N examined	% prevalence (95% CI)	Intensity of infection (sd)
By community			
Abang	83	28.9 (20.3–39.4)	22.4 (6435.4)
Akometam	89	22.5 (15.0–32.2)	481.8 (8841.9)
Ngat-Bane	99	30.3 (22.1–30.3)	446.5 (3945.4)
By sex			
Males	131	37.4 (29.6–45.9)	2594.7 (6035.6)
Females	140	17.9 (12.4–25.0)	1294.0 (7160.9)
By age group			
5–19	24	4.2 (0.7–20.2)	289.2 (1416.6)
20–34	69	23.2 (14.8–34.4)	1127.5 (3559.5)
35–49	40	30.0 (18.1–45.4)	3622.5 (10,562.8)
50-Over	138	32.6 (27.3–43.0)	2111.7 (6811.8)
Overall	271	27.3 (22.3–32.9)	1922.7 (6623.2)

*CI* confidence interval, *sd* standard deviation

### Intensity of infection

**Fig. 1:**
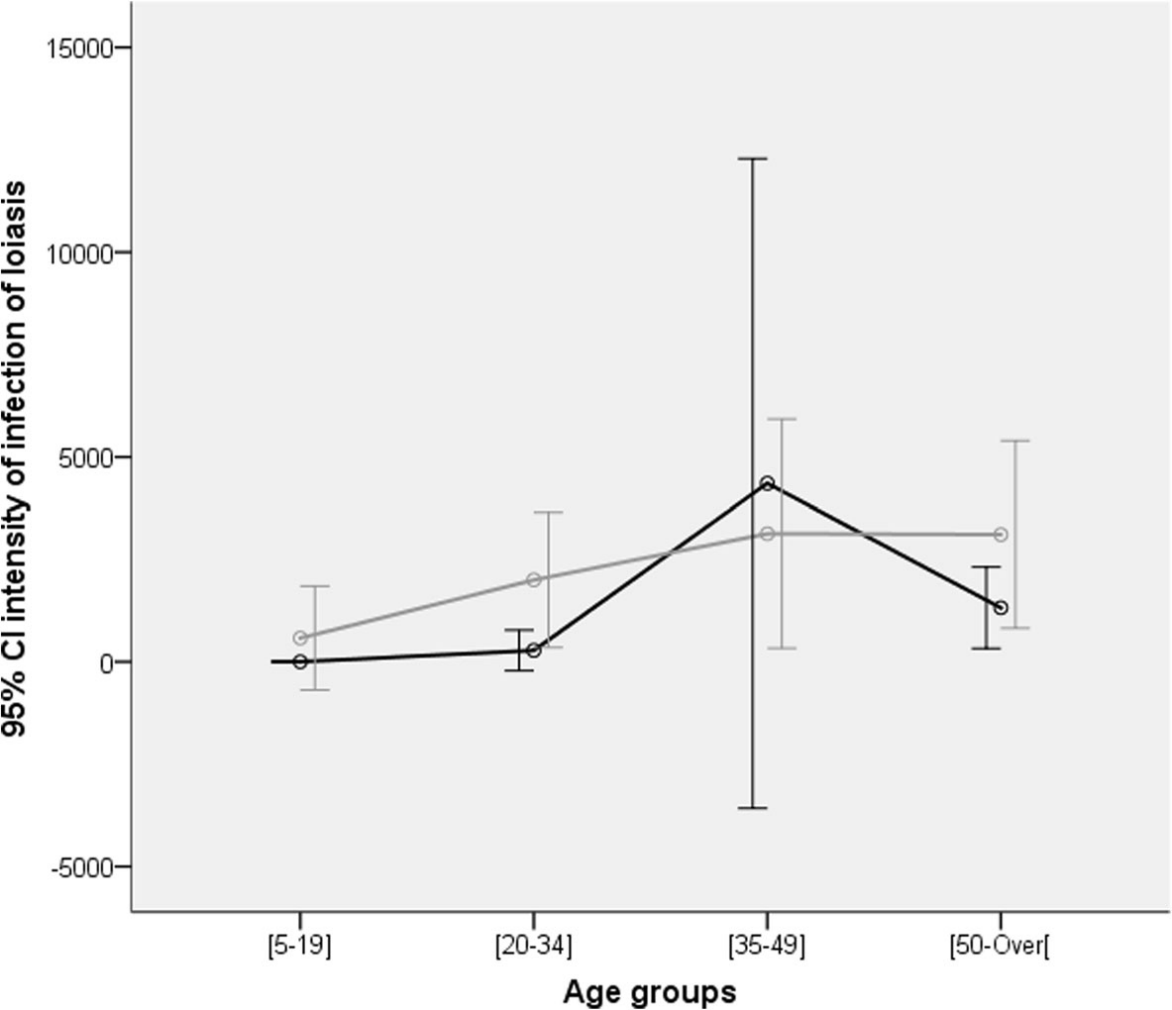
Trends in Loa microfilariaemia according to age groups in the Mbalmayo health district. Grey lines and bars represent males and dark lines and bars represent females

Regarding the intensity of infection, the mf density arithmetic mean of was 1922.7 (sd: 6623.2; maximum: 59,540) mf/mL. Like prevalence, intensity of infection was similar between communities (*p* = 0.528), whereas a high heterogeneity was reported between genders and age groups (Table 1). The intensity of Loa infection was higher among males (*p* < 0.0001) and a convex in form trends was observed among age groups (*p =* 0.032). The maximum microfilarial load was reached in individuals aged 35 to 49 years old, both in males and females (Table 1; Fig. 1). Besides these observations, 7.4% (7.7% amongst females) and 1.5% (1.9% amongst females) of enrollees harbored microfilarial loads exceeding 8000 mf/mL and 30,000 mf/mL, respectively, with similar trends between males and females (*p* > 0.816).

### Morbidity associated with loiasis

Among the 177 individuals to whom the standardized questionnaire was administered (the interview was not performed in the Akometam community for logistic constrains) 22.5% (95% CI: 15.0–32.2) and 26.0% (95% IC: 20.1–32.9) declared having already experienced Calabar swellings and sub-conjunctival migration of adult Loa, respectively. The prevalence of these clinical signs was similar among communities, genders, and age groups (*p* > 0.085) except for eye worm whose history was mostly recorded among individuals aged 21 years and over (*p* = 0.012). A positive association was found between the prevalence of loiasis (evaluated by microfilariaemia) and history of eye worm (OR: 5.250; 95% IC: 2.545–10.829; *p* < 0.0001) and Calabar swelling (OR: 5.039; 95% IC: 2.382–10.656; *p* < 0.0001).

### 23-years change in *Loa loa* endemicity

**Fig. 2:**
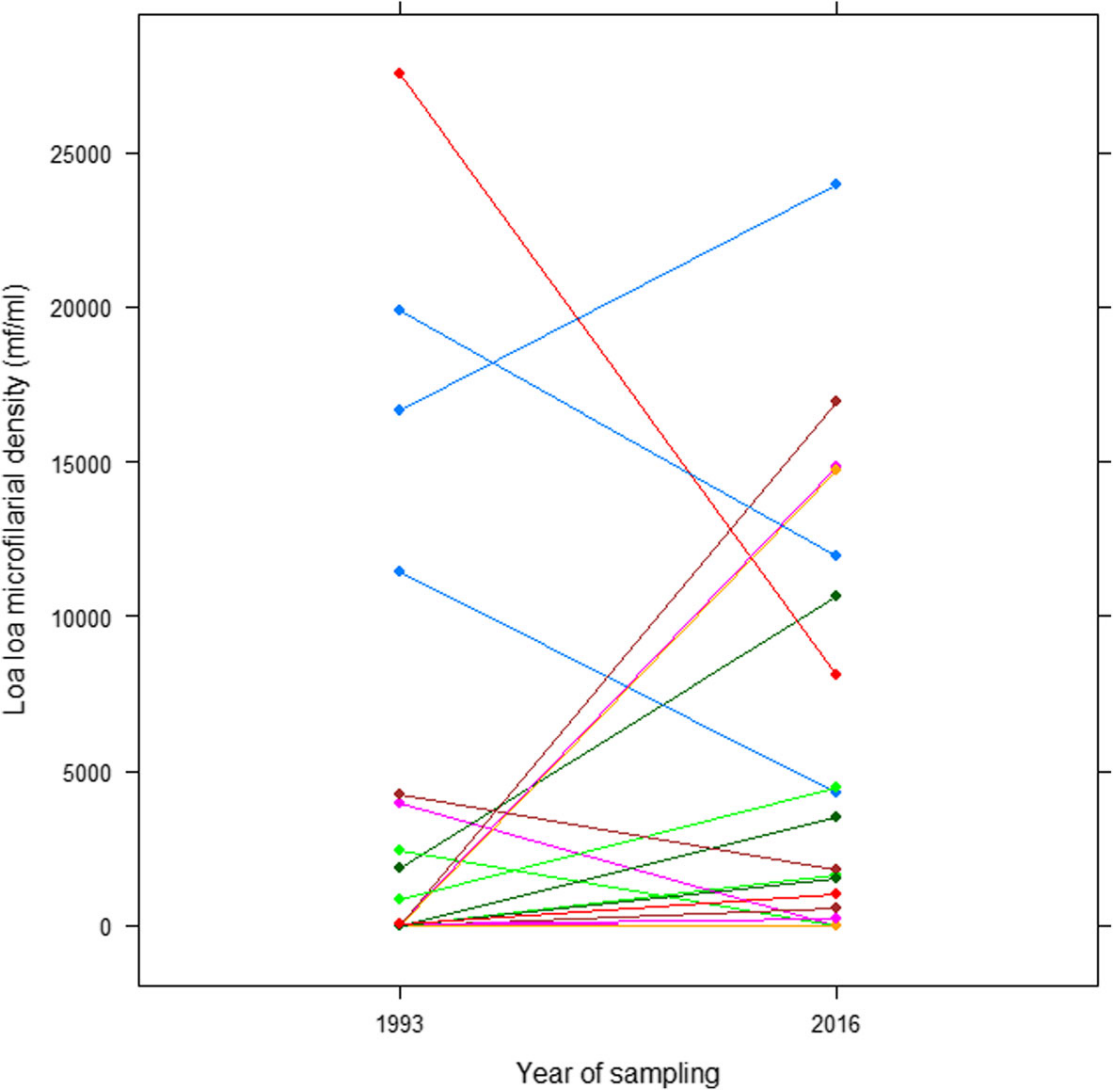
Individual microfilarial load trends between 1993 and 2016 in the Mbalmayo health district

Among the 271 individuals tested in 2016, 39 (of whom 27 males) were already present in 1993. A positive association was observed between the prevalence of Loa microfilariaemia and the duration of residence in the community (OR: 1.016; 95% CI: 1.005–1.027; *p* = 0,003). The infection rate, when considering only those 39 individuals, was relatively higher in 2016 (41.0% (95% CI: 27.1–56.6) than in 1993 (35.9%; 95% CI: 22.7–51.6), but the difference was not significant (Chi-square: 0.22; *p* = 0.639). Microfilarial densities were also similar between 1993 (mean: 2280.5; sd: 6074.0) and 2016 (mean: 3080.0; sd: 5872.3), though a slight increase was observed in 2016 (Wilcoxon signed ranks tests, *p* = 0.351) (Fig. 2). Importantly, 50% of heavily infected (microfilarial loads exceeding 8000 mf/mL) enrollees already exhibited high microfilarial densities 23 years ago.

## Discussion

This repeated cross-sectional study was carried out to investigate the long-term trends in prevalence and intensity of *Loa loa* infection in an area that has never benefitted from community preventive chemotherapy. The overall prevalence of loiasis was 27.3% in the three targeted communities; a figure similar to what was previously observed in the same communities [13–15] and indicating that this area is highly endemic for African eye worm [20]. The prevalence of the African eye worm, estimated by history of either migration of adult worm under the bulbar conjunctiva or the Calabar swelling, was not accurately predicted as previously demonstrated [18, 21]. Several reasons can explain this discrepancy: (i) in the present study, the interview was quicker and lighter (no image was shown) than in previous study; (ii) it was demonstrated that people presenting with loiasis associated clinical signs (migration of adult worm under the bulbar conjunctiva and the Calabar swelling) rarely harbor *L. loa* mf [22, 23]; (iii) finally the sample size of our study was small (only 177 individuals were interviewed for logistical constraints), thus reducing the power of statistical tests, and the likelihood to accurately capture this relationship. However, a positive association was found between Loa microfilarial prevalence and histories of both migration of eye worm and Calabar swelling.

The mean intensity of infection was 1922.7 (sd: 6623.2; maximum: 59,540) mf/mL, with 7.4 and 1.5% of enrollees harboring microfilarial loads exceeding 8000 mf/mL and 30,000 mf/mL, respectively. The relationship between prevalence and intensity of infection observed in the present study fits with previous observations [20]. Indeed, it was demonstrated that prevalence of Loa microfilariaemia between 20 and 30%, can predict approximately 5–9% of adults harboring microfilarial loads exceeding 8000 mf/mL, and 1–3% of adults with > 30,000 mf/mL [20]. Since severe adverse events have been correlated with high Loa microfilarial loads [10], these results (i) indicate that the implementation of ivermectin treatments in the Mbalmayo health district needs to be done with caution, and (ii) confirm why CDTI is yet to be implemented in this health district [11].

*Loa loa* prevalence and intensity of infection were found to increase while age was increasing (OR: 1.018; 95% CI: 1.005–1.032; *p* = 0.007). A convex in form trend was observed among age groups, the maximum microfilarial load being reached in individuals aged 35 to 49 years old (Table 1; Fig. 1). These results might indicate that the exposure to loiasis is increasing with age, the disease appearing to accumulate in infected individuals. In case of accumulation of the parasite consecutive to new infective bites by the vector, one would have expected an increase in prevalence and intensity of infection over time, particularly in the absence of any intervention targeting either the parasite or the vector. However, the convex in form profile displayed in the relationship between age and intensity of infection might suggest that this accumulation is somehow relative and might reverse in advanced age group. Similar results have been reported in previous studies, suggesting a noncumulative nature of this filarial disease [14]. Such observations have also been described in *Onchocerca volvulus* [24], another filarial nematode closely related to *L. loa*, and was explained by the fact that in younger individuals the parasite population has not reached its equilibrium, the number of maturing adult worms in these individual hosts exceeding that of dying worms. On the contrary, in older individuals the number of dying adult worms exceeds that of maturing worms as a consequence to their reduced exposure to chrysops’ bites because of their indoor habit related to their age. This latter hypothesis supports the decreasing trend in intensity of infection observed in advanced age.

A follow up of individuals already examined 23 years ago have shown that both the rate and intensity of infection remained almost unchanged (Fig. 2), indicating that the transmission of loiasis is stable over time. This stability in *L. loa* transmission was already observed in Gabon [25], Congo [26], and Cameroon (same site like in our study) [14] over a shorter interval (1–3 years). The hypotheses underpinning this stability were suggested to be inherent both to the parasite (mf production by adult worms) and to the host (immune defense mechanism). This long-term stability observed in *L. loa* infection is consistent with a noncumulative status of the disease observed in the present study. A 13-year follow up of loiasis transmission in the Lékié division (Central Cameroon), has also revealed a stability in entomological indices, including (but not limited to) chrysops infection and potential infective rates [27]. Despite these interesting findings, the sample size of this follow-up study was small (only 39 individuals examined in 1993 were found in 2016), and one can question representativeness and the generalizability of this small sample with regards to the general population. It is worth mentioning that similar observations have been previously reported elsewhere, even though within a shorter time frame, suggesting that these findings and associated interpretations are not unique to our study.

## Conclusions

This 23-year repeated cross-sectional study confirmed that the prevalence and intensity of *Loa loa* infection is stable over time, indicating that this filarial disease might be noncumulative [28]. It has been argued that infection with *Loa loa* can hinder the success of onchocerciasis and lymphatic filariasis control and/or elimination programs as a consequence of post-ivermectin SAEs. Given that this study has demonstrated that loiasis might be noncumulative and microfilarial densities are somehow stable over time, an increased risk of post-ivermectin SAEs in these untreated communities is unlikely or not expected, and the caution with which mass drug administration has to be implemented in loiasis endemic areas will not fundamentally change.

## Abbreviations

CDTI: Community directed treatment with ivermectin
CI: Confidence interval
mf/mL: Microfilariae per milliliter of blood
OR: Odds ratio
SAE: Severe adverse event
sd: Standard deviation
